# P-616. Impact of Implementing Rapid Molecular Testing Algorithms For Respiratory Virus Detection At A Pediatric Hospital in Kansas City

**DOI:** 10.1093/ofid/ofaf695.829

**Published:** 2026-01-11

**Authors:** Dithi Banerjee, Brian R Lee, Rangaraj Selvarangan

**Affiliations:** Children's Mercy Hospital, Overland Park, KS; Children's Mercy Kansas City, Kansas City, Missouri; Children’s Mercy Hospital, Kansas City, Missouri

## Abstract

**Background:**

Rapid molecular testing is crucial in pediatric care for the timely detection of respiratory viruses. Our aim was to determine the impact of a testing algorithm by comparing single-plex assays - ID NOW™ INFLUENZA A & B 2 and ID NOW™ RSV (Abbott) and a 4plex assay - Xpert®XpressCoV-2/Flu/RSV (FLUVID) for Flu A/B and RSV detection during the respiratory season of 2024-25.

**Table 1.**
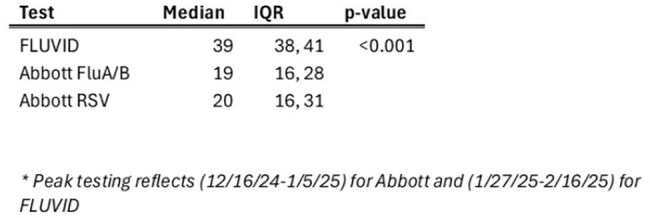
Turn-around-Time (minutes) during Peak Testing*, by test type

**Table 2.**
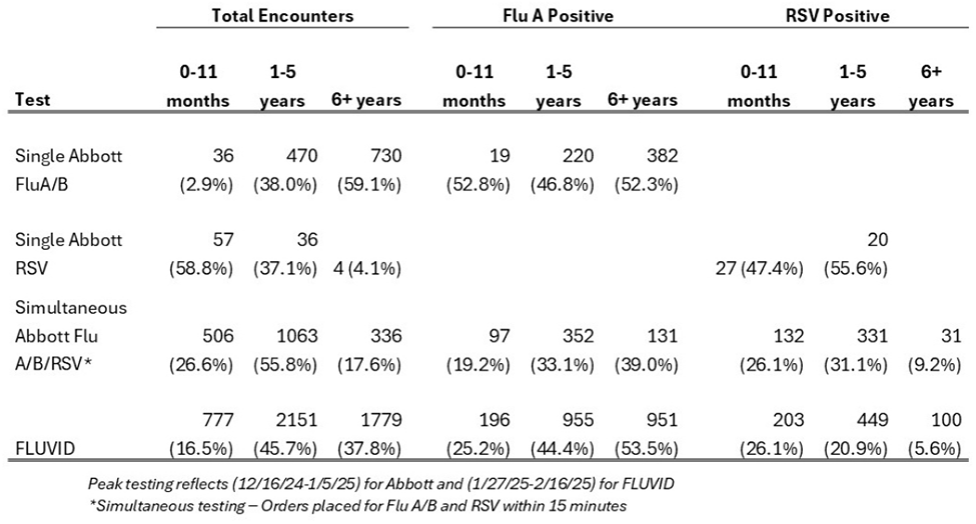
Viral Positivity during Peak Testing, by Test Type and Age Group

**Methods:**

Between November 25^th^ and December 31^st^, 2024, we offered the stand-alone Abbott assays (Flu A/B and RSV) for outpatient respiratory testing and switched to the 4-plex FLUVID assay from January to March 2025. Electronic health records of all children with an order for respiratory testing (Abbott or FLUVID) during this time frame and peak testing season (3 consecutive weeks of ≥1000 tests for respective assay) were analyzed to determine viral positivity, TAT, length of stay (LOS), and prescription for antivirals and antibiotics.

**Table 3.**
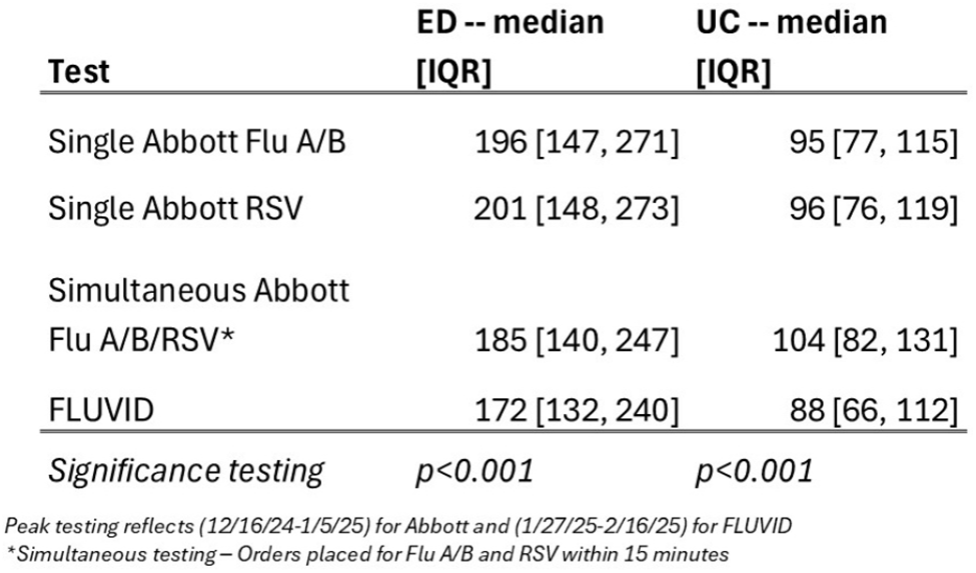
LOS in minutes during Peak Testing, by Location and Test Type

**Results:**

Overall, 6650 Abbott and 8100 FLUVID tests were done in 14 weeks; viral positivity ranged from 25% to 63%. During peak testing, median TATs were 19 minutes, 20 minutes and 39 minutes reported for Abbott Flu A/B, Abbott RSV and FLUVID assays, respectively (Table 1). All 3 assays showed >90% test completion within a 60-minute metric at the Emergency Department (ED) setting; however, FLUVID failed 8.5% of the times in the Urgent Care (UC). High positivity ( >46%) was reported with single-plex testing for Flu A and RSV across all age groups (Table 2). However, positivity was lower when physicians offered FLUVID or simultaneous testing for Flu A/B and RSV by Abbott (Table 2). Overall, LOS was shorter both in the ED and UC with FLUVID (Table 3). Higher proportion of children with Abbott Flu A/B tests (12.2%) received Baloxavir compared to those with FLUVID (5.2%).

**Conclusion:**

Our testing algorithm with a combination of single-plex and 4plex assays showed shorter TAT with Abbott but longer LOS with FLUVID. Providers opted for both types of tests when given a chance and reported higher positivity with Abbott assays. Age-appropriate children with Abbott Flu results received higher rate of Baloxavir at the time of discharge. However, all results may have been impacted by staffing, number of modules, and volume of testing and may account for the differences at each location.

**Disclosures:**

Brian R. Lee, PhD, MPH, Merck: Grant/Research Support Rangaraj Selvarangan, PhD, Altona: Grant/Research Support|Biomerieux: Advisor/Consultant|Biomerieux: Grant/Research Support|Biomerieux: Honoraria|Cepheid: Grant/Research Support|Hologic: Grant/Research Support|Hologic: Honoraria|Meridian: Grant/Research Support|Qiagen: Grant/Research Support

